# Lung Diffusion Capacity in Patients With Bilateral COVID-19 Pneumonia: A Three-Month Follow-Up Study

**DOI:** 10.7759/cureus.58897

**Published:** 2024-04-24

**Authors:** Marina Vasilj, Kristina Galic, Tanja Zovko, Gordana Kraljevic, Nikolina Pravdic, Belma Saric-Zolj, Marija Goluza Sesar, Danijel Pravdic

**Affiliations:** 1 Department of Lung Diseases, University Clinical Hospital Mostar, Mostar, BIH; 2 Department of Neurology, University Clinical Hospital Mostar, Mostar, BIH; 3 Clinic for Internal Diseases, University Clinical Hospital Mostar, Mostar, BIH

**Keywords:** covid-19 infection, diffusion capacity of the lungs for carbon monoxide, lung function test, respiratory function, pneumonia

## Abstract

Objectives: The aim of this study was to determine the short-term consequences of coronavirus disease 2019 (COVID-19) infection on pulmonary diffusion in patients with severe (but not critical) and moderately severe COVID-19 pneumonia during three months after COVID-19 infection.

Methods: A prospective study included 81 patients with an RT-PCR-test confirmed diagnosis of COVID-19 infection treated in the COVID Department of Lung Diseases of University Clinical Hospital Mostar. Inclusion criteria were ≥18-year-old patients, COVID-19 infection confirmed using real-time RT-PCR, radiologically confirmed bilateral COVID-19 pneumonia, and diffusion capacity of the lungs for carbon monoxide (DLCO) one and three months after COVID-19 infection. The pulmonary function was tested using the MasterScreen Body Jaeger (Jaeger Corporation, Omaha, USA) and MasterScreen PFT Jaeger (Jaeger Corporation, Omaha, USA) according to American Thoracic Society guidelines one and three months after COVID-19 infection.

Results: Forced vital capacity significantly increased three months after COVID-19 infection compared to the first-month control (p<0.0005). Also, a statistically significant increase in the FEV1 value (p<0.0005), FEV1%FVC ratio (p<0.005), DLCO/SB (p<0.0005), DLCO/VA value (p<0.0005), and total lung capacity (TLC) (p<0.0005) was observed in all patients.

Conclusion: Our study showed that recovery of DLCO/VA and spirometry parameters was complete after three months, while DLCO/SB was below normal values even after three months. Therefore, one month after the COVID-19 infection patients had partial recovery of lung function, while a significant recovery of lung function was observed three months after the COVID-19 infection.

## Introduction

Emerging viral diseases have become a major public health problem [1]. Coronavirus disease 2019 (COVID-19) is disease caused by the SARS-CoV-2 [2,3]. During various stages, symptomatology of COVID-19 is different. The most frequent clinical symptoms are fatigue, shortness of breath, cough, fever, and sputum production [4,5].

The health consequences of COVID-19 remain largely unclear [6]. The literature reports that patients may have persistent impairment in diffusion capacities after hospitalization [7]. Different pathophysiological events include diffuse alveolar epithelium destruction, hyaline membrane formation, capillary damage and bleeding, alveolar septal fibrous proliferation, and pulmonary consolidation [8].

Pulmonary function tests (PFTs), such as spirometry, diffusion capacity, and lung volumes, are mostly used for functional respiratory evaluations [9,10]. Chest computed tomography (CT) abnormalities may cause pulmonary fibrosis and should be analyzed together with lung function [11,12]. Patients who have severe COVID-19 pneumonia should be evaluated with full PFTs 12 weeks after hospitalization [13]. The first analysis of lung function showed that patients have a restrictive defect and a small airway dysfunction that can be persistent and not related to the disease severity [14]. The number of studies on the frequency and relevant predictors of COVID-19-related pulmonary sequelae is still limited with varying results [15].

Thus far, studies have not clearly determined the health consequences of COVID-19 patients. We designed our prospective study to investigate the prevalence of disturbances in the values of spirometric parameters and disturbances in diffusion parameters in post-COVID-19 patients and to investigate recovery of pulmonary function in these patients. This research might contribute to a better understanding of the impact of COVID-19 infection on respiratory function in the early recovery period to prevent long-term outcomes.

## Materials and methods

Study design and study population

The prospective study was conducted in the period from 2021 to 2022 and included patients with COVID-19 infection treated at the COVID Department of Lung Diseases of the University Clinical Hospital Mostar (UCH Mostar), Cantonal Hospital Livno, and Cantonal Hospital, Dr. Safet Mujić“ Mostar. Subjects were ≥ 18-year-old patients diagnosed with COVID-19 infection. Criteria for inclusion of patients were aged over 18 with severe unstable, but not critical, COVID-19 disease (The Modified Early Warning Score, MEWS 3-4) and moderately severe stable disease (MEWS ≤ 3) with comorbidity, according to the guidelines issued by the World Health Organization (WHO) in 2020 [16]. Severe unstable, but not critical illness (MEWS 3-4) was defined as illness with clinical or laboratory signs of the impaired ratio of arterial partial pressure of oxygen to fraction of inspired oxygen, with breathing shortness, dyspnea, tachypnea, need for oxygen supplementation >4 liters per minute for regaining oxygen saturation measured by pulse oximetry above 92%, but without critical signs (impairment of consciousness, septic shock) [3]. Diffusion capacity of the lungs for carbon monoxide (DLCO) using a single-breath technique method adjusted for hemoglobin was performed one month and three months after COVID-19 infection. According to the Global Lung Function, we classified patients with normal DLCO (≥80% predicted) or altered DLCO (<80% predicted) [17]. Exclusion criteria were patients with a history of chronic lung disease or severe lung comorbidities (such as pulmonary resection, pulmonary embolism, pre-existing interstitial lung disease) and patients incapable of completing pulmonary functional tests due to any reason. This clinical study was approved by the Ethics Committee of the UCH Mostar where the study was conducted (no. 1494/23). Our study was performed following the ethical standards of the Helsinki Declaration of 1975, as revised in 2000.

Data collection

We evaluated patients during hospitalization treatment for COVID-19 infection and three months after the infection in a nine-month period. The pulmonary function test was done in the Lung Function Laboratory of UCH Mostar. The MasterScreen Body Jaeger (Jaeger Corporation, Omaha, USA) was used for performing spirometry and MasterScreen PFT Jaeger (Jaeger Corporation, Omaha, USA) was for performing diffusion capacity of the lungs for carbon monoxide (DLCO) according to the American Thoracic Society guidelines one and three months after COVID-19. We analyzed the following parameters: FVC (forced vital capacity), FEV1 (forced expiratory volume in one second), FEV1/FVC (forced expiratory volume/forced vital capacity), DLCO/SB (single-breath diffusing capacity of the lung for CO), DLCO/VA (diffusing capacity for carbon monoxide divided by the alveolar volume), and TLC (total lung capacity) at first-month control. The patients came for follow-up after three months and the same parameters were analyzed again.

Statistical analysis

For this work, differences in the average values of individual parameters that describe the state of the disease at the time of admission and the state at the follow-up examination after three months were tested with the statistical T-test of dependent samples. For this test, data matching with a normal distribution was tested. The Shapiro-Wilk test was used to test normality. Statistical analyses were performed using IBM SPSS Statistics for Windows, Version 20 (Released 2011; IBM Corp., Armonk, New York, United States).

## Results

We included 81 subjects, aged from 27 to 89 years. The average age was 60 years, 45 men (56%) and 36 (44%) women. Patients were treated at the COVID Department of Lung Diseases of the UCH Mostar and in other hospitals. The number of patients treated in other hospitals (Cantonal Hospital Livno and Cantonal Hospital, Dr. Safet Mujić“ Mostar) was 22 (27%), while 59 (73%) patients were admitted to our department for hospital treatment (Table 1). 

**Table 1:** Demographic and clinical characteristics of the study population

Place of treatment	Sex	N	%	Median age	Maximum age	Minimum age
Other hospitals	Male	14	17	56	77	31
	Female	8	10	60	83	26
University Clinical Hospital Mostar	Male	31	38	64	84	33
	Female	28	35	65	81	25

**Figure 1:**
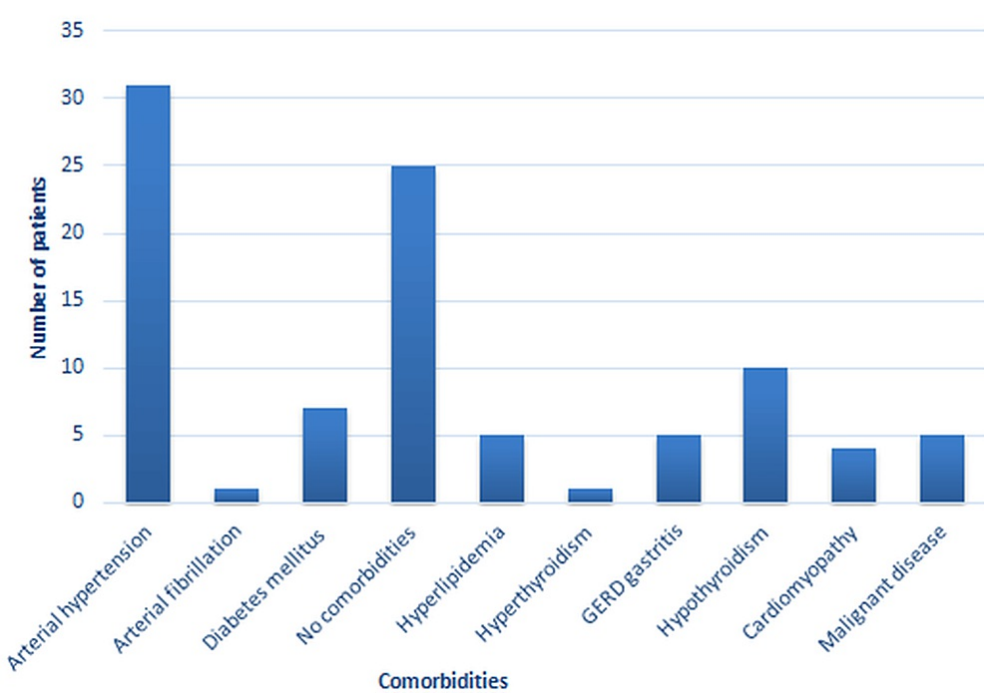
Prevalence of comorbidities in patients GERD: Gastroesophageal reflux disease

The number of smoking patients in the sample was 31%. That is, 25 patients were active smokers or ex-smokers at the time of admission for examination. The frequency distribution of comorbidities in patients in the sample is given in Figure 1.

The difference between the FVC level in the patients from the first-month control and the FVC level after the three-month control was tested using a t-test of dependent samples. According to the results of the t-test, a statistically significant increase in the value of FVC was determined from the admission of the patient (M=89.4; SD=17.0) to the control after three months (M=95.0; SD=17.1), t (80)=-6.44; p<0.0005 (Table 2).

**Table 2:** Values of the tested parameters at the first-month and three-month control FVC: forced vital capacity; FEV1: forced expiratory volume in first second; FEV1/FVC: forced expiratory volume/forced vital capacity; N: the size of the sample; M: mean deviation; SD: standard deviation; t: the Student's t distribution; df: the degrees of freedom; p: p-value

Spirometry parameter	Point of measurement	N	M	SD	t	df	p
FVC	first-month	81	89.4	± 17.0	-6.44	80	.000
	three-month control	81	95.0	±17.1			
FEV1	first-month	81	94.2	±19.2	-4.25	80	.000
	three-month control	81	98.2	±19.8			
FEV1/FVC	first-month	81	85.3	±8.6	3.38	80	.001
	three-month control	81	83.4	±8.5			

According to the results of the t-test for the FEV1 parameter, it can be concluded that there was a statistically significant increase in the FEV1 value from the first-month control of the patient (M=94.2; SD=19.2) until control after three months (M=98, 2; SD=19.8), t(80)=-4.25; p<0.0005 (Table 2).

The patients' FEV1/FVC ratio was analyzed from the first-month control (M=85.3; SD=8.6) and at the control after three months (M=83.4; SD=8.5). The results of the t-test of dependent samples showed that the decrease in the value of the tested FEV1/FVC ratio was statistically significant t(80)=3.38; p<0.005 (Table 2).

The dependent samples t-test was used to test the difference between the DLCO/SB level from the first-month control and the DLCO/SB level after three months. According to the results of the t-test, a statistically significant increase in the value of DLCO/SB was determined from the first-month control (M=63.2; SD=17.3) to the control after three months (M=73.0; SD=18.8), t (80)=-7.65; p<0.0005 (Table 3).

**Table 3:** Results of pulmonary function tests on first-month and three-month control DLCO/SB: single-breath diffusing capacity of the lung for CO; DLCO/VA: diffusing capacity for carbon monoxide divided by the alveolar volume; TLC: total lung capacity; N: the size of the sample; M: mean deviation; SD: standard deviation; t: the Student's t distribution; df: the degrees of freedom, p: p-value.

Diffusion parameter	Point of measurement	N	M	SD	t	df	p
DLCO/SB	first-month	81	63.2	±17.3	-7.65	80	.000
	three-month control	81	73.0	±18.8			
DLCO/VA	first-month	81	78.1	±16.6	-6.10	80	.000
	three-month control	81	85.6	±17.6			
TLC	first-month	81	84.5	±15.5	-4.62	80	.000
	three-month control	81	88.5	±13.9			

The dependent samples t-test was used to test the difference between the DLCO/VA level during the first months of control and the DLCO/VA level after three months. According to the results of the t-test, a statistically significant increase in the DLCO/VA value was found from the first-month control (M=78.1; SD=16.6) to the control after three months (M=85.6; SD=17.6), t(80)=-6.10; p<0.0005. Our study showed that recovery of DLCO/VA was complete after three months, and DLCO/SB was below normal values even after three months (Table 3).

The TLC value of the patients was analyzed during the first-month control (M=84.5; SD=15.5) and at the control after three months (M=88.5; SD=13.9). The results of the t-test of dependent samples showed that the increase in the value of the tested TLC was statistically significant t(80)=-4.62; p<0.0005 (Table 3).

## Discussion

In the prospective study of 81 subjects, we analyzed short-term consequences on pulmonary function in patients for three months after COVID-19 infection.

The patients surviving COVID-19 are frequently reported to have pulmonary sequelae [17,18]. The previous reports show that patients may have persistent impairment in respiratory function [19]. The first reports on lung function in these patients showed a restrictive defect and a small airway dysfunction that can be persistent and not related to the severity of the disease [14]. Authors reported diffusion capacity impairment followed by restrictive ventilatory defects, which are connected with disease severity [8]. In the international guidance on the management of COVID-19, 60% of experts were in favor of routine post-hospital PFT within 30-60 days regardless of the disease severity [18]. A few descriptive reports showed a substantial prevalence of altered DLCO percentage in survivors [19-21]. Pulmonary function, particularly DLCO, declined in COVID-19 survivors [22]. Patients with DLCO impairment have a higher percentage of interstitial pulmonary damage. Also, DLCO% performed four months after COVID-19 was the most important, independent correlate of more severe initial disease [11].

The critical period for diffusion capacity recovery is the first three months. This can be connected with capillary component damage recovery and accessible alveolar volume reduction [23]. Data from a study by Qin et al. pointed out that 44 (54%) of 81 patients had abnormal DLCO three months after COVID-19 (<80% predicted) [24]. Our study shows early recovery of pulmonary function in the first three months. The DLCO/SB was 63.2% at discharge and significantly improved to 73.0% at three months. The transfer coefficient of the lung for carbon monoxide (KCO)% pred significantly increased from 78.1% at discharge to 85.6% at three months. Our study showed that recovery of DLCO/VA was complete after three months, and DLCO/SB was below normal values even after three months. The change rates of DLCO% pred and KCO% were significantly higher in 0-3 months. It is important to address whether the DLCO assessment, in addition to spirometry, can be considered in routine clinical follow-ups of COVID-19 patients [24]. Early lung rehabilitation and individualized approach to recovery are worthy of additional investigation [25]. Lung function tests may be regarded as indispensable tools for monitoring functional impairment, planning rehabilitation, managing complications, and preventing long-term outcomes [26].

The limitation of our study it is a single-center study. Also, the initial values of the pulmonary functional tests were not available because the patients did not perform the tests upon discharge due to the severity of the disease, or due to epidemiological measures. Pulmonary function data before COVID-19 infection are lacking even though patients with chronic respiratory diseases were excluded from the study.

## Conclusions

Patients with COVID-19 pneumonia significantly recover their lung function in the first month after COVID-19 infection. The parameter that did not recover even after three months was DLCO/SB, suggesting that disease effects on lung diffusion might be the limiting recovery factor in COVID-19 patients. Early monitoring is important in predicting the long-term consequences of COVID-19 pneumonia on lung function.
